# Public attitudes towards consent for the donation of surplus frozen eggs to research

**DOI:** 10.1093/humrep/deag007

**Published:** 2026-02-03

**Authors:** J Langford, J Demaree-Cotton, M Johnston

**Affiliations:** School of Medicine, The Faculty of Medicine, Nursing and Health Sciences, Monash University, Clayton, Australia; The Uehiro Oxford Institute, University of Oxford, Oxford, UK; Monash Bioethics Centre, School of Philosophical, Historical and Indigenous Studies, Faculty of Arts, Monash University, Clayton, Australia

**Keywords:** ethics, egg freezing, egg donation, surplus eggs, egg disposition, egg storage, consent, consent model, public attitudes, survey

## Abstract

**STUDY QUESTION:**

In the context of donating surplus frozen eggs (SFE) to research, what level of information disclosure, and associated consent model, do the public believe most effectively allows donors to make an informed decision, exercise autonomy, and be treated morally?

**SUMMARY ANSWER:**

The public supports the information disclosure requirements of both a specific and broad consent model in this context, with the latter considered to better enhance autonomy and facilitate the moral treatment of SFE donors.

**WHAT IS KNOWN ALREADY:**

Despite research indicating that many individuals’ first preference is to donate their SFEs to research, donation rates remain low. One possible reason for this is the way consent processes for the donation of SFEs to research are currently regulated, specifically that their high information requirements limit opportunities to donate. There is a notable lack of research on how consent processes should operate, and more specifically, how much information a person should be provided before providing consent, in the context of donating SFEs to research.

**STUDY DESIGN, SIZE, DURATION:**

An online experimental survey of 225 participants was conducted. The survey assessed the impact of two variables—Information Disclosure and Preference Fulfilment—on participants’ views towards whether a consent process allowed for informed, autonomous consent and the moral treatment of donors.

**PARTICIPANTS/MATERIALS, SETTING, METHODS:**

A nationally representative sample of the UK public was recruited using the online platform Prolific. The survey consisted of a vignette-based experimental design, one free-text question, and demographic data collection. Quantitative data were summarized using descriptive statistics and the relationship between variables was tested using ANOVAs and *t*-tests, where appropriate. Inductive content analysis through manual coding was performed on the free-text question.

**MAIN RESULTS AND THE ROLE OF CHANCE:**

Participants considered both specific and broad information disclosure as sufficient for informed consent (mean Consent Judgements *M *= 6.49/7 and *M *= 5.79/7, respectively). The ability to fulfil disposition preferences was critical to the public’s assessment of whether a consent process enabled donors to act autonomously and be treated morally. Participants agreed that a potential donor was able to make an autonomous decision if their preference to donate their SFEs to research was fulfilled (mean Autonomy Judgement *M *= 5.46/7, mean Moral Judgement *M *= 5.63/7), but not when it was not (mean Autonomy Judgement *M *= 3.96/7, mean Moral Judgement: *M *= 4.76/7).

**LIMITATIONS, REASONS FOR CAUTION:**

Ecological validity of online surveys is limited, and data may be subject to response biases. Additionally, the sample size was relatively small. Finally, since the sample population was based in the UK, the generalizability of the survey findings to other countries may be limited.

**WIDER IMPLICATIONS OF THE FINDINGS:**

Our findings underscore the need to review and possibly update consent processes for the donation of SFEs to research. We encourage policy discussion in light of our findings, specifically the consideration of a shift towards a broad consent model. Doing so may allow more donors to fulfil their disposition preference, facilitate the movement of SFEs out of storage, and respond to the shortage of eggs currently available for research.

**STUDY FUNDING/COMPETING INTEREST(S):**

This research was supported by The British Academy (grant number KF8\230096). The survey component of this study was funded by the Uehiro Oxford Institute. M.J. has received research funding from Monash IVF and Ferring Pharmaceuticals. She reports honorarium and travel support from Gideon Richter.

**TRIAL REGISTRATION NUMBER:**

N/A.

## Introduction

Egg freezing is becoming increasingly popular (Johnston *et al.*, 2021; Human Fertilisation & Embryology Authority, 2023a). However, most individuals who freeze their eggs will never return to use them (Gürtin *et al.*, 2019; Bahadur, 2021; Blakemore *et al.*, 2021; Walker *et al.*, 2022; Johnston *et al.*, 2024). Eggs may be frozen for fertility preservation, or as part of infertility treatment (e.g. if no sperm was available on the day of egg collection, or in preparation for procedures such as microTESE). Regardless of the reason for egg freezing, all individuals with surplus frozen eggs (SFEs) will face decisions regarding their disposition. In many jurisdictions, individuals with SFEs must decide between three disposition options: disposal of SFEs, donation of SFEs to others for reproductive purposes, or donation of SFEs to approved biomedical research (Caughey *et al.*, 2023; Human Fertilisation & Embryology Authority, 2023b). Despite research indicating that many individuals’ first preference is to donate their SFEs to research (Caughey *et al.*, 2021), the number of SFEs donated to research remains low, or even non-existent (Johnston *et al.*, 2024). Scholars suggest that this is a missed opportunity (Friedrich, 2020; Johnston *et al.*, 2024), as frozen eggs are an important research resource (Delange, 2022; Human Fertilisation & Embryology Authority, n.d.), and the demand for their use in medical research is high (Fuscaldo *et al.*, 2023). Moreover, SFE storage is becoming an increasingly prevalent public issue globally (Rinehart, 2021), given the number of eggs rapidly accumulating in storage facilities (Johnston *et al.*, 2024). It is possible that the storage crisis could be alleviated if individuals were able to fulfil their donation preference more readily.

One potential barrier to the donation of SFEs to research is the way that informed consent processes are currently regulated (Johnston *et al.*, 2024). In the UK, alongside other jurisdictions like Australia, current guidelines require all relevant information to be disclosed about the specific project to which an individual is consenting to donate their eggs (Mayor, 2007; National Health and Medical Research Council, 2017). This reflects the requirements of a *specific consent model* (Caulfield and Kaye, 2009). One implication of this model is that it creates a tension between maintaining high level information disclosure and facilitating the fulfilment of preferences to donate, as the very requirement for specificity restricts opportunities for donation. Under this model, individuals cannot consent to the donation of their SFEs to future, undefined research projects, as the information disclosure requirements of a specific consent model cannot be met. Therefore, a person’s ability to donate to research is contingent on whether a project is actively recruiting at the time eggs are relinquished from storage. As these situations are limited, eggs that could have been directed to research may instead be discarded (Johnston *et al.*, 2025).

Given the research demands for four eggs, and people’s desires to donate, scholars have recently suggested easing the current rigorous consent requirements to open more opportunities to donate (Johnston *et al.*, 2025). One such proposal is moving towards a *broad consent model* (Mills *et al.*, 2024; Fuscaldo *et al.*, 2025). Broad consent models are increasingly employed in genomic research, as it is rarely possible at the time of sample collection to foresee all potential future uses of genomic samples. Broad consent models impose lower information requirements; prospective donors are provided information about the general area of research that their sample may be used in, but specific study details are not disclosed (Wiertz and Boldt, 2022). This lack of specificity opens up more opportunities to donate, as it is possible to donate to future, unspecified research (Hansson *et al.*, 2006; Sheehan, 2011; Steinsbekk *et al.*, 2013; Grady *et al.*, 2015). Existing empirical research suggests that the public largely accepts the use of a broad consent model in the context of biomaterial donation to research (Hansson *et al.*, 2006; Simon *et al.*, 2011; Grady *et al.*, 2015; Garrison *et al.*, 2016; Warner *et al.*, 2018). However, attitudes towards a broad consent model in the context of donating SFEs to research have not yet been explored.

There is a notable lack of research on how consent processes should operate, and more specifically, how much information a person should be given before providing consent, in the context of donating SFE to research. Our study aims to address this gap by examining public attitudes towards informed consent in the context of donating SFE to research, specifically in a UK context. Members of the UK public were surveyed to capture baseline attitudes on consent models in this context, given the acknowledged importance of public trust and engagement in supporting legitimate and ethical regulatory frameworks (Gille *et al.*, 2024).

We focus specifically on one central element of informed consent: information disclosure, as this is widely recognized as a key criterion of valid consent (Beauchamp, 2009). First, we assess public views on the ability of different levels of information disclosure to allow for informed consent. We then examine the tension between information disclosure and preference fulfilment to understand which the public believes should be prioritized when they come into conflict. To do so, we assess the impact of both information disclosure and preference fulfilment on moral evaluations of the donation process and on the perceived autonomy of donors. In the context of this study, preference fulfilment refers to people donating their eggs to research. However, we acknowledge in practice, patients may have other disposition preferences.

This study provides the first account of attitudes towards information disclosure and preference fulfilment, offering important evidence to inform future discussions and policy reviews on informed consent for the donation of SFEs to research.

## Materials and methods

This study consisted of an online survey (Supplementary Data File S1) hosted on Qualtrics, which followed a vignette-based experimental design. Before distributing the final survey, a pilot study was conducted on a cohort of 33 individuals, and adjustments were made to the survey where necessary.

### Study design

**Figure 1.**
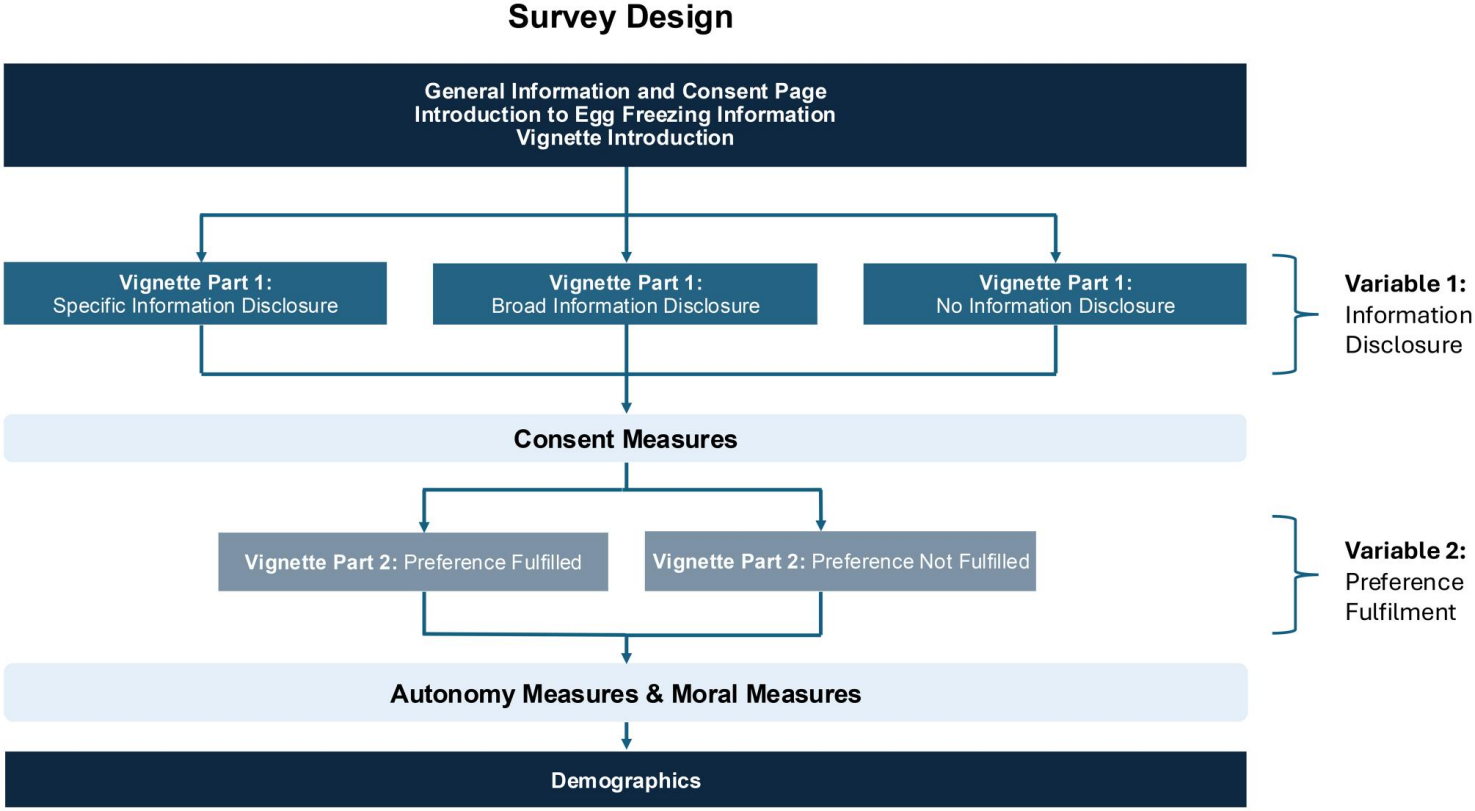
**Survey design summary chart.**  *Summary Chart representing the flow of survey design and order of survey items*.

In our study, Participants were randomly assigned to read one of six vignettes, in a 3 (Information Disclosure*;* Specific Information, Broad Information, No Information) by 2 (Preference Fulfilment; Preference Fulfilled, Preference Not Fulfilled) between-subjects design (Fig. 1). The survey design was informed by similar studies on lay reasoning about consent (e.g. Demaree-Cotton and Sommers, 2022).

### Study population

A power analysis was conducted in G*Power 3.1, which indicated that at least 64 participants would be required in each Information Disclosure group, to achieve 80% power to detect statistically significant differences at *P*<0.05 between conditions, assuming a medium effect size (*d* = 0.5). To ensure our sample reflected public views across different demographics, a sample of 300 participants representative of the UK population in terms of gender, age, and ethnicity based on data from the UK Office of National Statistics (Prolific, 2024) was recruited using Prolific, an online recruitment platform. A UK sample was recruited due to the relevance of this ethical issue in the UK. Participants were reimbursed at a rate of £13.71/h (for a median 6.34-min duration) upon completion of the survey.

### Procedure and materials

The survey started with a participant information sheet, after which participants were asked to give their consent to participate in the survey. All participants were then given information about egg freezing, before being presented with a vignette (differing according to the Information Disclosure and Preference Fulfilment conditions to which they had been assigned).

The introduction section of the vignette was identical for all participants and described a hypothetical situation involving Jane, a woman with SFEs, whose disposition preference was to donate them to research. Following this, Part 1 of the vignette described how much information Jane received about the research to which she would be donating her SFEs. Participants received different versions depending on their assigned Information Disclosure condition (Table 1). All participants were then told ‘*Jane decides to proceed and consent to donating her frozen eggs to research.*’ Next, participants were asked whether Jane was able to make a (i) valid, (ii) sufficiently informed, and (iii) free choice to donate her eggs to research (referred to as Consent Measures from here on) (Table 2). After the first Consent Measure, participants were asked ‘Please tell us why you answered as you did to the above question’ via free-text.

**Table 1.** Vignette part 1.

Information disclosure condition	Vignette manipulation
No information	*Jane is told she can donate her eggs to medical research. There are studies currently running and in need of donor eggs. But she is told nothing about the nature or quality of the research.* *No information is provided to Jane about the studies that will use her eggs if she donates them. She is not provided with information about the general area of research, nor is she given information about the research objectives, how her eggs will be used, the risks and benefits of the studies, and who is conducting the research.*
Specific information	*Jane is told she can donate her eggs to approved medical research. This means the research will be conducted safely, fairly, and responsibly. There are approved studies currently running and in need of donor eggs.* *Jane is provided full and detailed information about the specific studies that will use her eggs if she donates them, such as their research objectives, how her eggs will be used, the risks and benefits of the studies, and who is conducting the research.*
Broad information	*Jane is told she can donate her eggs to approved medical research. This means the research will be conducted safely, fairly, and responsibly. There are approved studies currently running and in need of donor eggs.* *Jane is provided with information about the general area of research that her eggs will be used for if she donates them. She is not given information relating to the specific studies that will use her eggs, such as their research objectives, how her eggs will be used, the risks and benefits of the studies, and who is conducting the research.*

**Table 2.** Consent measures.

Measure category	**Statements** *
Consent measures	a) ‘Jane gave valid consent to the donation of her eggs to research’ b) ‘Please tell us why you answered as you did to the above question’ ‘Jane’s decision to donate her frozen eggs to research was sufficiently informed’ ‘Jane made a free choice to donate her frozen eggs to research’

* *Participants responded to each statement on a 7-point Likert scale where 1 = Strongly Disagree and 7 = Strongly Agree, except for 1 b) which allowed open text responses. Questions 1a and 1 b were presented to participants first, and then the order of 2 and 3 was randomized*.

On the next page of the survey, participants were presented with Part 2 of the vignette, which described whether Jane’s preference to donate her SFEs to research was fulfilled or not. Participants received a different version depending on their assigned Preference Fulfilment condition (Table 3). Subsequently, further agreement statements were presented to participants, assessing views towards Jane’s autonomy (Autonomy Measures) and her moral treatment during the consent process (Moral Measures) (Table 4). All agreement statements were collected on a labelled 7-point Likert scale (1 = *Strongly Disagree*; 2 = *Disagree*, 3 = *Somewhat Disagree*, 4 = *Neither Agree nor Disagree*, 5 = *Somewhat Agree*, 6 = *Agree*, 7 = *Strongly Agree*). Statements would therefore be considered to indicate participant agreement if their rating given was ≥5.

**Table 3.** Vignette part 2.

Preference fulfilment condition	Vignette manipulation
Preference Fulfilled	After making the decision to donate her eggs to research, Jane receives confirmation that her frozen eggs have been donated to research.
Preference Not Fulfilled	However, after making the decision to donate her eggs to research, Jane is informed that due to technicalities in current donation regulations, she is unable to donate her frozen eggs to research after all. With no other options, Jane discards her frozen eggs.

**Table 4.** Autonomy measures and moral measures.

Measure category	Statements*
Autonomy measures	‘Jane’s autonomy was respected’. ‘Jane’s control over her frozen eggs was undermined’. *(reverse-coded)* ‘Jane was unable to make a decision in line with her preferences and values’. *(reverse-coded)*
Moral measures	‘Jane was treated fairly’. ‘Jane was treated wrongly’. *(reverse-coded)*

* *Participants responded to each statement on a 7-point Likert scale where 1 = Strongly Disagree and 7 = Strongly Agree. Whether participants were presented with Autonomy or Moral Measures first was randomized, as was the order of the questions within both Measure categories*.

Finally, participants answered demographic questions. To ensure that participants understood the content of the vignettes, were paying attention, and responding correctly to instructions survey, two comprehension checks and one attention check (see Supplementary Data File S1) were included.

### Statistical analysis

Statistical analysis was performed on the quantitative data using IBM SPSS Statistics (version 29.0.2.0, UK). The data were directly exported from Qualtrics into IBM SPSS Statistics, to minimize personal error associated with manual data entry. Descriptive statistics measuring ranges, frequencies, central tendencies (means), and SDs were applied to the quantitative survey items. For negatively phrased statements, reverse coding was applied. For the agreement statements, Cronbach’s alpha scores were used to assess the scale reliability of Consent, Autonomy, and Moral Measures, respectively. The measures were averaged to give one overall Judgement score if Cronbach’s alpha was 0.7 or above for the respective Measure categories. Two-way between-subject ANOVAs were conducted to assess the impact of Information Disclosure and Preference Fulfilment on a participant’s Consent Judgements Autonomy Judgements and Moral Judgements, followed by independent samples *t-*tests for pairwise comparisons. A *P*-value of <0.05 was considered significant. For the qualitative free-text question, inductive content analysis through manual coding was performed to identify important response categories (Vears and Gillam, 2022).

### Ethics and open science

This study was granted ethics approval from the University of Oxford Social Sciences and Humanities Interdivisional Research Ethics Committee (Reference Number: R806912/RE016) and was ratified by the Monash University Human Research Ethics Committee (Project Number: 42701). Methods, sampling procedures, and analysis plans were pre-registered at: osf.io/k7qjz.

## Results

### Demographics

Three hundred Prolific users took the survey. Participants who failed one or more checks were excluded from further analysis (N = 75), leaving a final sample of 225 participants (Table 5).

**Table 5.** **Participant demographics.** *

Characteristic	Breakdown	Frequency (%)
Age	18–24	23 (10.2%)
	25–34	41 (18.2%)
	35–44	38 (16.9%)
	45–54	38 (16.9%)
	**55–64**	**53 (23.6%)**
	65–74	27 (12%)
	75+	5 (2.2%)
Sex	**Female**	**117 (52.0%)**
	Male	105 (46.7%)
	Prefer not to say	3 (1.3%)
Education	GCSE’s, O-Levels or equivalent	38 (16.9%)
	A Levels, IB or equivalent	53 (23.6%)
	**Bachelor’s degree (e.g. BA, BSc)**	**97 (43.1%)**
	Post-graduate degree (e.g. MA, MSc, PhD)	31 (13.8%)
	Other	5 (2.2%)
	Prefer not to say	1 (0.4%)
Nationality	**UK/British (including dual citizen)**	**206 (91.6%)**
	Other	14 (6.2%)
	Prefer not to say	5 (2.2%)
Marital status	Single	61 (27.1%)
	Partnered	51 (22.7%)
	**Married**	**107(47.6%)**
	Other	2 (0.9%)
	Prefer not to say	4 (1.8%)
Biological children	**Has had biological children**	**137 (60.9%)**
	Has not had biological children and **does not** want to in the future	40 (17.8%)
	Has not had biological children and **does** want to in the future	23 (10.2%)
	Has not had biological children and **is not sure** if wants to in the future	21 (9.3%)
	Prefer not to say	4 (1.8%)
Use of assisted reproductive technologies (ART)**	Egg freezing	2 (0.9%)
	Other ART	9 (4.0%)
	**None**	**212 (94.2%)**
	Prefer not to say	4 (1.8%)
Self-rated socio-economic status (SES) level (on a scale of 1–10)	1	8 (3.6%)
	2	8 (3.6%)
	3	21 (9.3%)
	4	25 (11.1%)
	5	50 (22.2%)
	**6**	**56 (24.9%)**
	7	47 (20.9%)
	8	7 (3.1%)
	9	0 (0.0%)
	10	0 (0.0%)
	Prefer not to say	3 (1.3%)
Religion	**No religion**	**134 (59.6%)**
	Christian	66 (29.3%)
	Muslim	10 (4.4%)
	Jewish	1 (0.4%)
	Buddhist	2 (0.9%)
	Other	2 (0.9%)
	Prefer not to say	10 (4.4%)

* *Modal responses are shown in bold text*.

** *Participants were able to select more than one answer for this question, responses are cumulative*.

### Consent judgements

**Figure 2.**
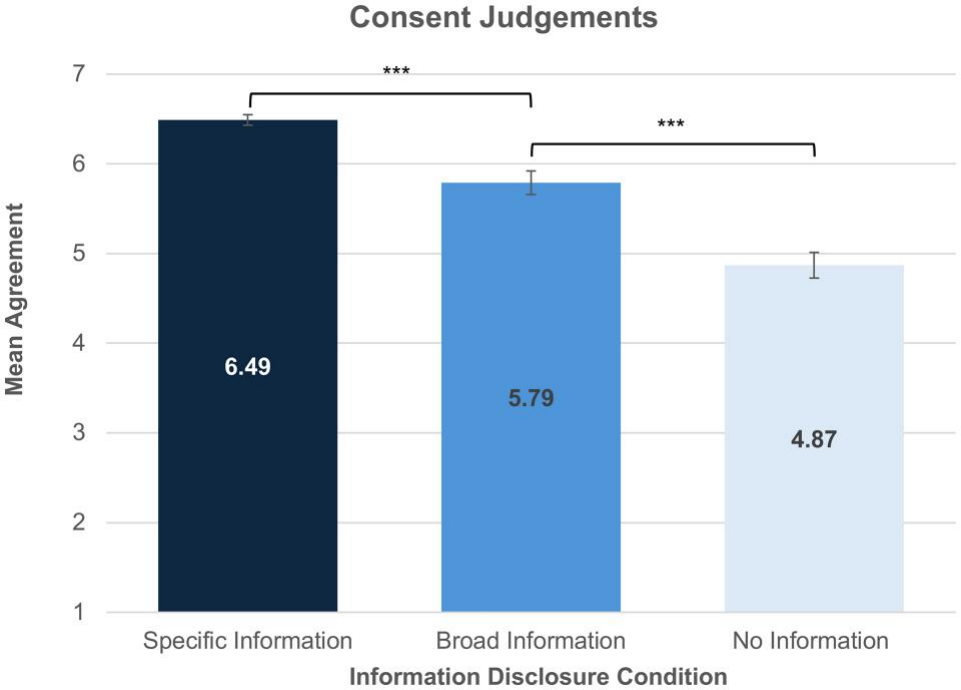
**Consent judgements.**  Error bars represent standard errors, *** indicates a significant difference between groups at P<0.001. Responses were gathered on a 7-point Likert scale where 1 = Strongly Disagree and 7 = Strongly Agree. Participants in both the Specific and Broad Information Disclosure groups agreed that consent was valid.

The three Consent Measures (see Supplementary Fig. S1) showed high scale reliability (α = 0.75), thus, a mean Consent Judgement score was utilized for further analysis. Information Disclosure significantly affected participants’ Consent Judgements (*F*(2, 219)=47.49, *P*≤0.001, $\eta_{P}^{2}$=0.30). Consent Judgements were significantly higher in the Specific Information condition (*M *= 6.49, SD = 0.50) than the Broad Information condition (*M *= 5.79, SD = 1.14) (*t*(103.87)=4.83, *P*<0.001, *d *= 0.78). Consent Judgements were also significantly higher in the Broad Information condition (*M *= 5.79, SD = 1.14) than the No Information condition (*M *= 4.87, SD = 1.25) (*t*(150)=4.74, *P*<0.001, *d *= 0.77) (Fig. 2).

Participants elaborated on whether they considered Jane’s consent to be valid using free text. Seven key content categories were identified: *Information Disclosure, The Act of Giving Consent; Autonomy Considerations, Sensitivity of Reproductive Material Donation; Type of Consent; Preferences; Benefit of Research* (Supplementary Table S1). In what follows, we outline the main reasons provided per information condition.

All 73 participants in the Specific Information condition agreed (i.e. scored 5 or above) that Jane offered valid consent. Many justified their answers by referring to *Information Disclosure*, specifically noting that Jane ‘knew what they [her eggs] would be used for’. Others cited *The Act of Giving Consent*, for example stating, ‘Jane consented, which is valid consent by definition’. *Autonomy Considerations* were also referenced, with one participant stating that ‘Jane made the decision on her own’, touching on Jane’s freedom from influence, and another emphasizing personal autonomy, stating that ‘[egg donation is a] personal choice’. Some participants also referenced *Preferences* and *Benefit of Research*, noting Jane’s wish to donate her SFEs to research and the potential for ‘scientific breakthroughs’.

Of the 76 participants in the Broad Information condition, 67 (88.2%) agreed that Jane gave valid consent. Many referred to *Information Disclosure* as the reason for their agreement, stating that Jane was ‘informed’, or that Jane was aware of the information disclosure limits and still chose to consent. *Autonomy Considerations* were also cited, with one participant explaining they believed Jane’s consent was valid, as the SFEs ‘were hers to decide what to do with’. Among the seven participants who disagreed (i.e. scored 3 or below), concerns focused on *Information Disclosure*, particularly that Jane was ‘insufficiently informed’ and ‘should’ve been given more detail’ about the research.

In the No Information condition, 55 of 76 (72.4%) participants agreed that Jane gave valid consent. Justifications again commonly referenced *Information Disclosure*, often pointing to Jane’s awareness of the limitations in the information provided. Some participants cited the *Type of Consent*, with one suggesting that Jane’s consent was valid as she gave her ‘consent in writing’*. Benefit of Research* was also mentioned, with participants describing eggs as a ‘valuable’ resource that ‘may help … others’. Of the 19 participants who disagreed that Jane gave valid consent, *Information Disclosure* was most frequently cited, with concerns that Jane was not ‘given the basic information’ needed for valid consent.

### Autonomy judgements

The three Autonomy Measures (see Supplementary Fig. S2) showed high scale reliability (α = 0.80), thus, a mean Autonomy Judgement score was utilized for further analysis. Autonomy Judgements were significantly higher in the Preference Fulfilled condition (*M *= 5.46, SD = 1.25), than in the Preference Not Fulfilled condition (*M *= 3.96, SD = 1.30) (*F*(1, 219)=75.40, *P*≤0.001, $\eta_{p}^{2}$=0.26).

**Figure 3.**
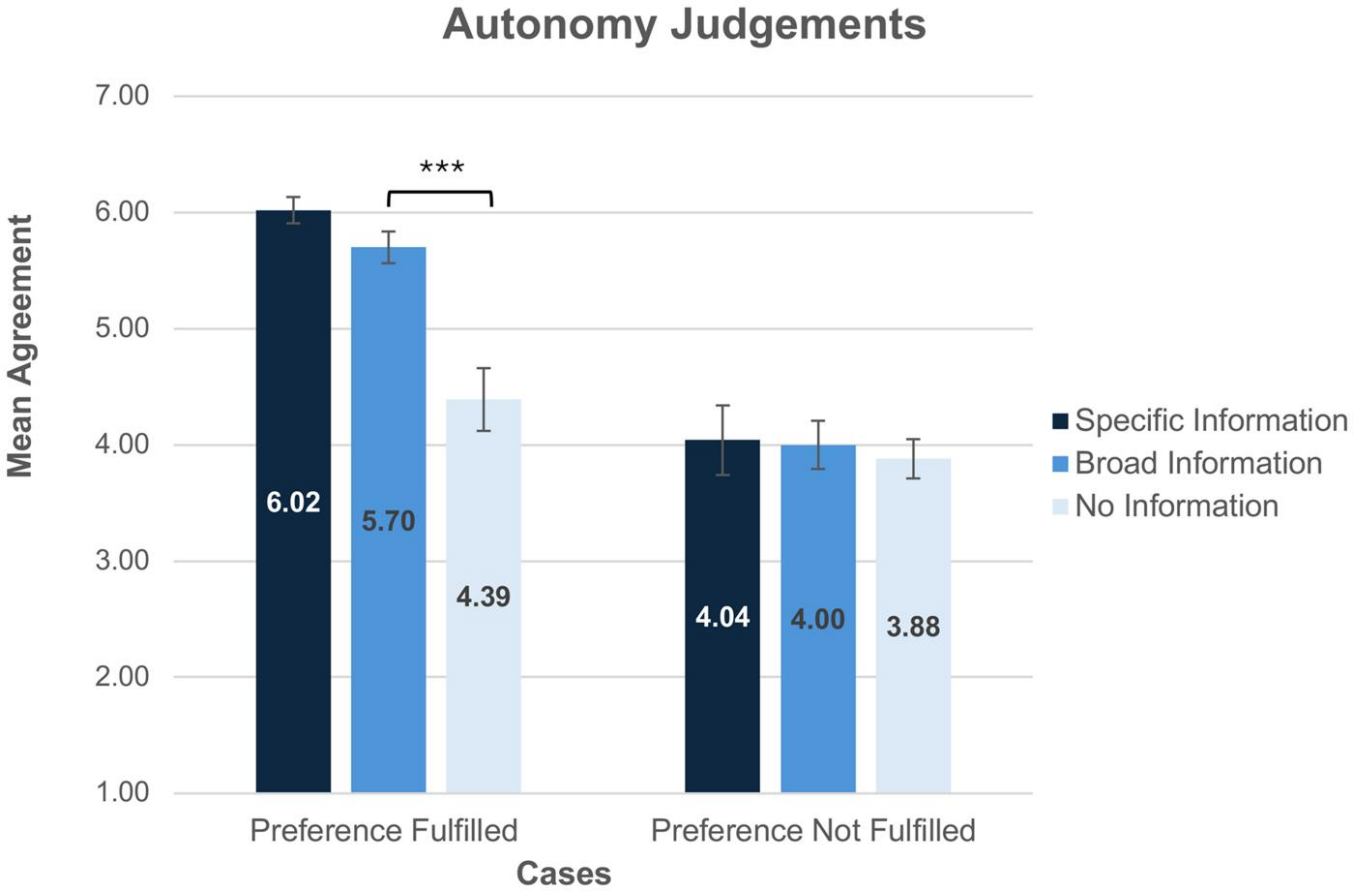
**Autonomy judgements.**  Error bars represent standard error, *** indicates a significant difference between groups at P<0.001. Responses were gathered on a 7-point Likert scale where 1 = Strongly Disagree and 7 = Strongly Agree. Across both the Specific and Broad Information groups, participants’ Autonomy Judgments were higher in the Preference Fulfilled group, and represented agreement that Jane was able to act autonomously.

Information Disclosure also significantly affected Autonomy Judgements (*F*(2, 219)=11.61, *P*≤0.001, $\eta_{p}^{2}$=0.10). But this main effect was qualified by a significant interaction between Information Disclosure and Preference Fulfilment (*F*(2, 219)=7.93, *P*≤0.001, $\eta_{p}^{2}$=0.07). Within the Preference Fulfilled condition, participants in the Broad Information condition had higher Autonomy Judgements than participants in the No Information condition (*M *= 5.70, SD = 0.80; *M *= 4.39, SD = 1.53), respectively; (*t*(46.14)=4.36, *P*<0.001, *d *= 1.01). However, there was no significant difference in Autonomy Judgements between the Specific Information condition (*M *= 6.02, SD = 0.77) and the Broad Information condition (*M *= 5.70, SD = 0.80) (*P *= 0.076). By contrast, in the Preference Not Fulfilled condition, there was no significant difference between the Specific Information condition (*M *= 4.04, SD = 1.56) and the Broad Information condition (*M *= 4.00, SD = 1.33) (*P *= 0.917), or the Broad Information condition (*M *= 4.00, SD = 1.33) and the No Information condition (*M *= 3.88, SD = 1.12) (*P *= 0.650) (Fig. 3).

### Moral judgements

The three Moral Measures (see Supplementary Fig. S3) showed high scale reliability (α = 0.90), thus a mean Moral Judgement score was utilized for further analysis. Moral Judgements in the Preference Fulfilled condition (*M *= 5.63, SD = 1.51) were significantly higher than in the Preference Not Fulfilled condition (*M *= 4.76, SD = 1.40) (*F*(1, 219)=18.673, *P*≤0.001, $\eta_{p}^{2}$=0.079).

**Figure 4.**
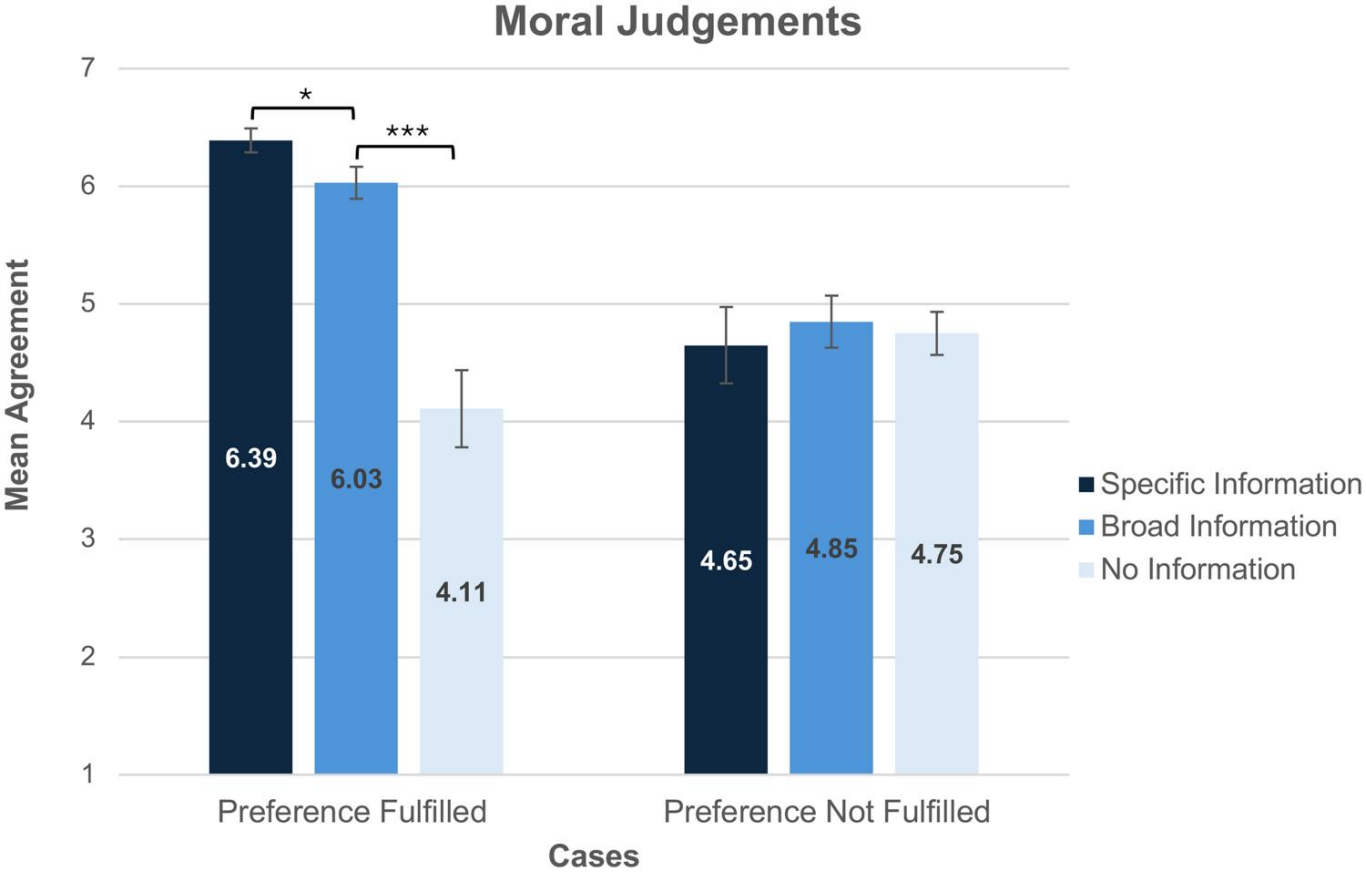
**Moral judgements.** Error bars represent standard error, * indicates a significant difference between groups at *P*<0.05, *** indicates a significant difference between groups at *P*<0.001. *Responses were gathered on a 7-point Likert scale where 1 = Strongly Disagree and 7 = Strongly Agree Across both the Specific and Broad Information groups, participants’ Moral Judgments were higher in the Preference Fulfilled group, and represented agreement that Jane was treated morally*.

Information Disclosure also significantly affected Moral Judgements *(F*(2, 219)=16.10, *P*≤0.001, $\eta_{p}^{2}$=0.13). However, this main effect was qualified by a significant interaction between Information Disclosure and Preference Fulfilment, *(F*(2, 219)=16.71, *P*≤0.001, $\eta_{p}^{2}$=0.13). In the Preference Fulfilled condition, Moral Judgements were significantly higher in the Specific Information condition (*M *= 6.39, SD = 0.69) than the Broad Information condition (*M *= 6.03, SD = 0.80) (*t(79*)=2.18 *P *< 0.032, *d *= 0.49). Moral Judgements were also significantly higher in the Broad Information condition (*M *= 6.03, SD = 0.80) than the No Information condition (*M *= 4.11, SD = 1.85) (*t(41.47*)=5.41, *P*<0.001, *d *= 1.37). By contrast, in the Preference Not Fulfilled condition, there was no significant difference in Moral Judgements between the Specific Information condition (*M *= 4.65, SD = 1.69) and the Broad Information condition (*M *= 4.85, SD = 1.42) (*P *= 0.589) or the Broad Information condition (*M *= 4.85, SD = 1.42) and the No Information condition (*M *= 4.75, SD = 1.21) (*P *= 0.71) (Fig. 4).

## Discussion

To our knowledge, this study is the first to report on public attitudes towards information disclosure and preference fulfilment within consent processes for the donation of SFEs to research. We report two key findings. First, both broad and specific information disclosure were regarded by the public as sufficient for informed consent. Second, fulfilling disposition preferences was critical to whether the public considered consent processes as able to support donor autonomy and moral treatment, irrespective of information disclosure levels. Given the increased opportunity for preference fulfilment under a model of broad consent, we argue that our findings offer preliminary grounds for moving away from current models of specific consent to a model of broad consent.

### Information disclosure

Our findings suggest that the public agree that both broad and specific information disclosure levels can facilitate valid consent in the donation of SFEs to research (mean Consent Judgements *M *= 5.79 and *M *= 6.49, respectively). Across both the Specific and Broad Information Disclosure groups, participants commented that enough information was provided for meaningful, informed consent. Specifically, participants in the Broad Information condition reasoned that Jane’s consent was valid as she ‘had the necessary information that she needed for her decision’. Furthermore, some suggested that because Jane was aware of the amount of information given to her, and that she consented knowing that this information was broad, her consent was valid. For example, one participant stated that Jane ‘…could have refused to donate [her eggs] if she felt she needed more information before consenting’.

By contrast, participants in the no information group tended not to agree, or have a strong view, on whether Jane was able to provide valid consent (*M *= 4.87). One participant explained: ‘I don’t feel that one can call this informed consent…[Jane] is totally in the dark about any associated risks’. This concept was echoed by another participant, who stated that Jane was unable to offer valid consent, as she had ‘not been given the basic information [required]’. These findings align with the ethical literature, which acknowledge that at least some degree of information disclosure is necessary for consent to be meaningful (Sheehan, 2011; Grady *et al.*, 2015). This ensures that consent is not merely procedural, but still reflects an individual’s capacity to make an autonomous and informed decision.

Notably, while participants in the No Information group were less likely to agree that Jane was able to make an informed consent decision, the mean response fell only slightly below the pre-determined agreement threshold of 5. These judgements are higher than expected, considering the normative significance of information disclosure for valid consent (Beauchamp, 2009). It is possible that these findings indicate that members of the public may place less weight on information than is typically assumed. Indeed, participants appear to have drawn on various considerations, often conflating the mere act or form of consent with its ethical validity. Some justified their agreement that Jane gave valid consent, by appealing to the fact that ‘she did give consent’ or that ‘she gave her consent in writing’. Yet, simply agreeing or signing a form does not in itself meet ethical standards of consent (Wynn and Israel, 2018).

A different possibility is not that participants completely lack a concept of consent that is sensitive to information provision, but rather that participants have more than one concept of consent. It is possible that people have two distinct concepts of consent: a superficial form, focused more on the act of saying ‘yes’, and a deeper form, reflecting normatively meaningful consent. This distinction may explain why participants rated Jane’s ability to consent higher than expected: their responses may have reflected their agreement that Jane consented in one, more superficial sense, even if they also believed that, in a deeper sense, Jane did not truly consent in a morally transformative way.

### Preference fulfilment

When participants were told that Jane was able to fulfil her preference to donate her SFEs to research, there was a high level of agreement that she acted autonomously, and was treated morally, provided she received either Specific or Broad Information about the research. Notably, these participants’ judgments of Jane’s autonomy and moral treatment did not differ greatly between the Specific and Broad information groups. In line with our earlier findings, when Jane was offered no information participants were less likely to agree that Jane acted autonomously or was treated morally.

While the public is largely supportive of both specific and broad information disclosure, this support is contingent on individuals having the opportunity to fulfil their donation preferences. Participants agreed that a potential donor was able to make an autonomous decision if their preference to donate their SFEs to research was fulfilled (mean Autonomy Judgement *M *= 5.46, mean Moral Judgement *M *= 5.63), but not when this preference was not satisfied (mean Autonomy Judgement *M *= 3.96, mean Moral Judgement *M *= 4.76). Notably, when the preference to donate was not fulfilled, participants’ Autonomy and Moral Judgements did not differ regardless of whether specific, broad, or no details about the research were provided. This suggests that in cases where an individual is unable to fulfil their preference to donate SFEs to research, the public sees no all-things-considered ethical benefit to increased information disclosure.

The high value placed on the ability for a consent model to accommodate preference fulfilment was expressed through the free-text responses. Participants raised that ‘Jane made her preference clear’ and that she ‘did not want her valuable eggs to be wasted’ as key considerations in whether her consent to SFE donation to research was valid. Notably, the qualitative responses in the survey were collected before the Preference Fulfillment condition had been introduced in the vignette, meaning participants raised the importance of preference fulfilment without prompting. This suggests the public independently prioritizes preference fulfilment in their assessment of the acceptability of a consent process.

Preference fulfilment is considered to be a key justification for informed consent processes, particularly in relation to promoting individual autonomy (Faden and Beauchamp, 1986; O’Neill, 2003; Taylor, 2009; Sheehan, 2011). Providing participants with information about research is said to respect autonomy by enabling them to make a decision about their participation that is consistent with their values, preferences, and desires (Faden and Beauchamp, 1986; Taylor, 2009; Sheehan, 2011; Koplin *et al.*, 2022). Some ethicists, however, have suggested that consent processes should not just *respect* autonomy, but actively *promote* it, such as through the fulfilment of preferences (Sullivan, 2016; Koplin *et al.*, 2022). It is important to note that not all individual preferences are relevant to autonomy, and should be fulfilled. Rather, preferences that are central to an individual’s core values or plans, especially those that relate to their own bodies, should be the ones which are promoted. Given that eggs may be viewed as a sensitive tissue type (Parker, 2011), and that people have genuine concern about their disposition outcome (Johnston *et al.*, 2025), we argue that, in the context of SFE donation to research, individual autonomy should be promoted, where possible. Accordingly, a broad consent model may be the most appropriate in this context, as individuals have more opportunity to act on their preference to donate their eggs to research.

A common criticism of a broad consent model is the loss of specific details about the research being consented to (Hansson *et al.*, 2006; Hofmann, 2009). However, while donors may not receive *all* information in broad consent situations, it does not mean they can’t make properly informed, and ethical, decisions—particularly if they consent with awareness of the kind of information that is lacking (Sheehan, 2011; Helgesson, 2012). As Sheehan (2011) states; by deciding to engage with a broad consent model, an individual has chosen to accept a lower level of information disclosure, and instead consent to defer future use decisions to others, which is still an ethical, autonomous, and informed choice. Our findings lend weight to this argument; participants agreed that individuals were able to provide valid consent, act autonomously, and be treated morally when provided with broad information about the research. Crucially, prioritizing preference fulfilment over exhaustive information disclosure does not undermine informed consent or respect for autonomy. By providing sufficient information while enabling individuals to act on their preferences, a broad consent model upholds autonomy and strengthens the ethical justification for its use in SFE donation.

### Future considerations

It is important to note that the practical implementation of a broad consent model in the context of SFE donation raises important questions that merit further exploration. These include concerns around whether the sensitive and non-regenerative nature of eggs should warrant more stringent consent requirements (Parker, 2011). Concerns may also arise about whether a broad consent model can accommodate diverse donor preferences and beliefs, particularly in relation to research applications that some may find objectionable (e.g. research involving stem cells or cloning). While we did not seek to address the practical question of implementing a broad consent model for SFE donation, we suggest several strategies that could respond to such concerns. These include establishing an oversight body to review and approve eligible research projects, allowing donors to opt-out of categories of research they find objectionable. Another consideration is the possibility of integrating a dynamic consent platform with a broad model of consent. As argued by Mills *et al.* (2024) and Fuscaldo *et al.* (2025), this would enable ongoing communication and the accommodation of evolving donor expectations. Future research could explore the ethical and practical viability of such suggestions.

### Strengths and limitations

A key strength of this study is that, to our knowledge, it is the first to assess public views on the matter of consent to SFE donation to research. As SFE storage is becoming a prevalent public issue globally (Rinehart, 2021), our findings offer timely insights. The data offer evidence-based preliminary recommendations that serve as a solid foundation for guiding future policy and ethical discussions.

While the use of an online experimental survey facilitates the collection of a sufficiently large number of responses to identify statistically significant response patterns, this method introduces potential limitations. These include a lack of control over the environment in which participants took the survey, and response biases. Of note, participants may have had different or limited understandings of certain terms used in the survey, such as ‘autonomy’ or ‘valid consent’, and this may have influenced their responses. Furthermore, a specific limitation of using the online platform Prolific to distribute the survey, is that it employs a nonprobability sampling method. Although quotas were set to match key demographic characteristics, participants self-select. While the advertisement of the survey did not reveal that it would concern consent or issues of preference fulfilment, it was listed as ‘Views on Human Frozen Egg Donation to Research’. This could introduce bias, as individuals who choose to participate may have strong feelings about the topic under investigation, which may limit the generalizability of the survey results.

Future research should explore the acceptability of a broad consent model among important stakeholder populations, such as fertility specialists or counsellors and those with SFEs in storage, to ensure that consent practices are ethically acceptable to the populations they are serving. This study’s findings could also be complemented by other qualitative research methods, such as interviews or focus groups, to allow for direct comparison and deeper discussion of different consent approaches in this context.

Additionally, our paper assessed public views on consent models in which participants were directly informed about the information they would or would not receive regarding the research to which their eggs would be donated (Table 2). Future research and policy discussions should consider what information potential donors should be explicitly told, including disclosing what they will not receive, to ensure that consent approaches are clearly defined, ethical, and publicly acceptable.

Finally, this study has focused on one key consideration in informed consent procedures: information disclosure. We acknowledge that other aspects of consent are also important to consider, for example, participant comprehension of the information given. Other considerations, like the competence of individuals donating their eggs, as well as how the information is provided to individuals with SFEs, are also crucial to the ethical assessment of consent processes and should be examined in future research.

## Conclusion

As the number of eggs being frozen increases (Johnston *et al.*, 2021), so too does the number languishing in storage (Bahadur, 2021; Rinehart, 2021; Johnston *et al.*, 2024). Yet, regulatory frameworks governing consent for the donation of SFEs to research have not evolved to adequately support donor autonomy and disposition preferences. This study provides the first empirical evidence of public views on consent processes in this context, specifically examining the ethical tension between information disclosure requirements and promotion of donor disposition preferences. Our findings indicate that while both broad and specific information disclosure are considered acceptable, preference fulfilment is critical for consent to be seen as autonomous and morally acceptable. These results provide preliminary support for reforming consent processes, specifically, through consideration of a broad consent model. Further research should explore key stakeholder perspectives and the feasibility of implementation. As the number of eggs being frozen continues to outpace those being used in reproductive treatment, it is incumbent that policy-makers consider the conditions under which egg disposition, specifically the option to donate, is managed. Continued efforts are required to ensure that individuals with SFEs are supported to make disposition decisions that align with their preferences and values.

## Supplementary Material

deag007_Supplementary_Data_File_S1

deag007_Supplementary_Figure_S1

deag007_Supplementary_Figure_S2

deag007_Supplementary_Figure_S3

deag007_Supplementary_Table_S1

## Data Availability

The data underlying this article will be shared on reasonable request to the corresponding author.
