# Supplementary material for: Public attitudes towards consent for the donation of surplus frozen eggs to research
Source: Hum Reprod. 2026 Feb 3;41(3):343–52. doi: 10.1093/humrep/deag007 (PMC13017042; doi:10.1093/humrep/deag007)
Supplement: deag007_Supplementary_Data_File_S1 [file deag007_supplementary_data_file_s1.docx]

**Supplementary Data File 1.** Views on Frozen Egg Donation to Research – Survey
