# Supplementary material for: Public attitudes towards consent for the donation of surplus frozen eggs to research
Source: Hum Reprod. 2026 Feb 3;41(3):343–52. doi: 10.1093/humrep/deag007 (PMC13017042; doi:10.1093/humrep/deag007)
Supplement: deag007_Supplementary_Figure_S2 [file deag007_supplementary_figure_s2.pdf]

Human Reproduction, 2026, 1–1
https://doi.org/10.1093/humrep/deag007
Supplementary Data

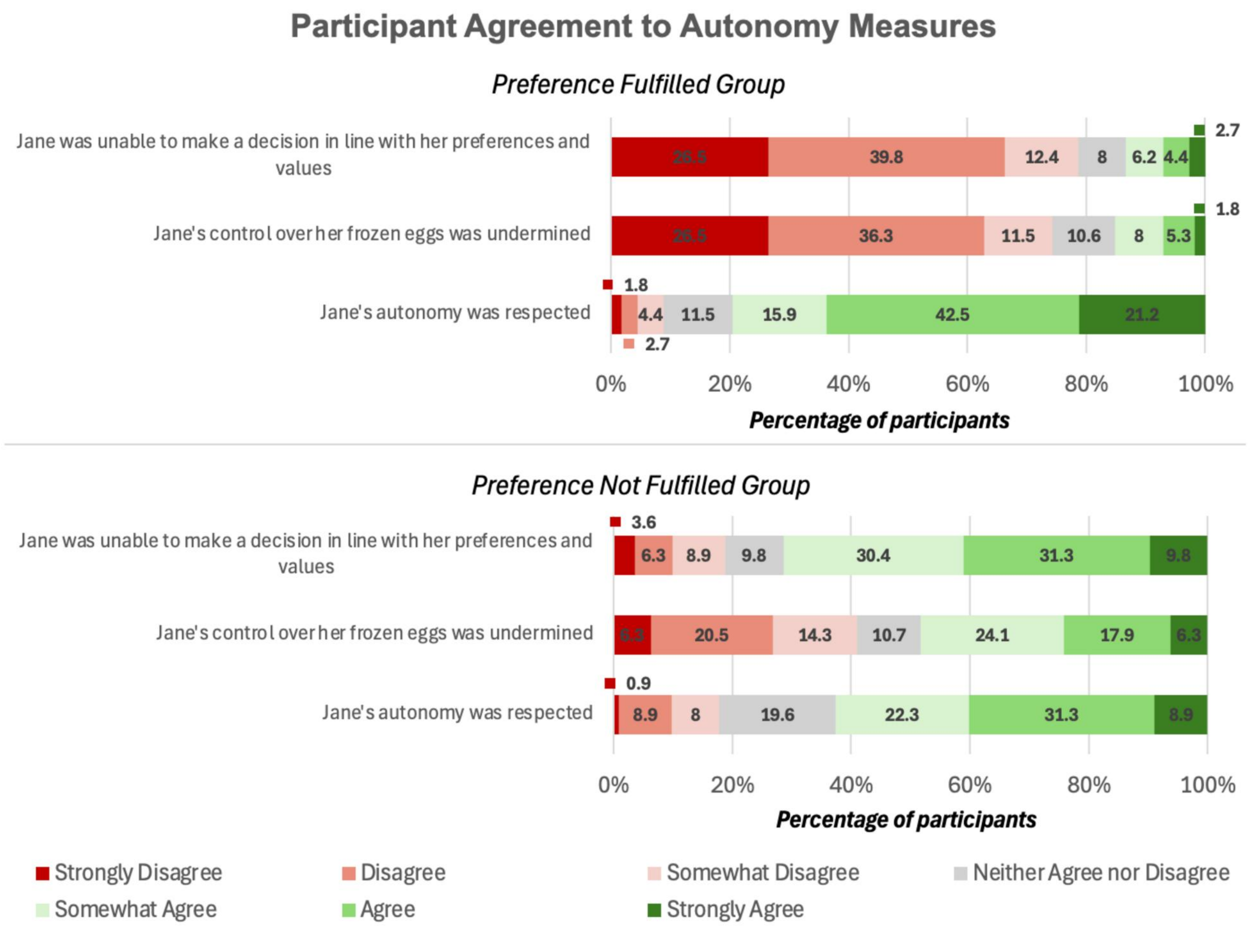

Supplementary Figure S2. Participant agreement to autonomy measures. Figures representing participant agreement to each of the three Autonomy Measures, split by Preference Fulfilment condition.
