# Supplementary material for: Public attitudes towards consent for the donation of surplus frozen eggs to research
Source: Hum Reprod. 2026 Feb 3;41(3):343–52. doi: 10.1093/humrep/deag007 (PMC13017042; doi:10.1093/humrep/deag007)
Supplement: deag007_Supplementary_Table_S1 [file deag007_supplementary_table_s1.pdf]

OXFORD
human reproduction
Human Reproduction, 2026, 1–2
https://doi.org/10.1093/humrep/deag007
Supplementary Data

Supplementary Table S1. Qualitative data on participant agreement that Jane gave valid consent to the donation of her frozen eggs to research.

Information disclosure condition	Agreement that Jane gave valid consent (number of participants)	Categories referenced	Exemplary quotes
**Specific Information**	**Agree*** (73)	Information Disclosure	• 'Jane received all of the information … which enabled her to make a well-informed decision … '<br>• 'Jane was informed of how her eggs would be used including the benefits and risks that come along with it'
		The Act of Giving Consent	• 'Jane consented, which is valid consent by definition'<br>• 'Because she did give consent'
		Autonomy Considerations	• 'It's personal choice and she chose what she wanted to do with her eggs'<br>• 'Jane made the decision on her own'
		Preferences	• 'Jane specifically told them that she wanted them to go to research and not to be used as donor eggs … '<br>• 'Jane did not want her eggs to go to waste, and she did not want to donate them for IVF … '
		Research Benefit	• 'To me it is common sense not to waste the eggs. Research can advance many scientific breakthroughs that could benefit mankind'
**Broad Information**	**Agree*** (67)	Information Disclosure	• 'Jane had the necessary information that she needed for her decision'<br>• '… she obviously received all the information she needed to make this decision'<br>• 'She was told how the eggs would be used so she was informed'.
		The Act of Giving Consent	• 'Because she agreed to do so'<br>• 'The story said she had consented'
		Autonomy Considerations	• 'They were hers to decide what to do with … '<br>• '… as far as i know [Jane] is mentally competent to make this decision'
		Preferences	• 'She didn't want her eggs to go to waste and she felt uncomfortable donating them to others'<br>• 'She decided to donate her eggs rather than them being used by someone else or being destroyed'
		Research Benefit	• 'Since I am happy if the donated eggs are used for useful research'.
	**Neither Agree nor Disagree**** (2)	Information Disclosure	• 'Firstly, I think Jane should've been given more detail with the scientific research … '<br>• '… she deserved to know the type of research the researcher would be carried out with her eggs'
		The Act of Giving Consent	• 'Jane gave consent for her eggs to be used for research … '
		Autonomy Considerations	• '… but if she didn't want or need the info then that's a person's own individual option'
	**Disagree***** (7)	Information Disclosure	• 'Because she wasn't given full details about the projects, their purpose and what would be done with her eggs'<br>• 'I feel for her consent to be valid she should have access to all the details of the research'
		Autonomy Considerations	• '… She may not realize there was more information to this or that she should ask for more information'
		Sensitivity of Reproductive Material Donation	• 'Very sensitive/emotional subject, full disclosure would prevent future thoughts along the lines of—I really wished I had asked … '<br>• 'Research purposes' is very broad—could there be any chances of the eggs being fertilized? If so, it

(continued)

Supplementary Table S1. (continued)

Information disclosure condition	Agreement that Jane gave valid consent (number of participants)	Categories referenced	Exemplary quotes
			would go against Jane's original wishes and be the thing she wanted to avoid'
**No Information**	**Agree*** **(55)**	Information Disclosure	• 'She knew what they would be used for and consented to that with the information given to her'<br>• 'I have gone through IVF and had to make this decision with similar levels of information'<br>• 'She still agreed to donate her eggs although she was not given, nor did she ask for more information'
		The Act of Giving Consent	• 'The last sentence states that Jane consents to donate her eggs to research'<br>• 'It stated in the text that she consented'
		Autonomy Considerations	• 'Its personal choice ... '<br>• ' ... she does not want to keep the eggs or pay for them anymore, so she, in essence, relinquished her ownership. She was presented with the different options available to her and she chose the one that suited her most ... '
		Type of Consent	• 'She gave her consent in writing'
		Preferences	• 'Jane didn't want the eggs to go to waste or to be used for another birth'<br>• 'She does not want her valuable eggs to be wasted'<br>• 'Her feeling of not wasting an important commodity'
		Benefit of Research	• 'Shortage of eggs. Research may help IVF for others'
	**Neither Agree nor Disagree**** **(2)**	Information Disclosure	• 'People should be allowed to choose to forgo receiving detailed information about the research before consenting to donate their eggs to research. I think Jane as an individual, made a conscious and balanced decision according to her own wishes; furthermore, this was done on the assumption that she knew the benefits and risks. However, I also don't think Jane received information to the fullest extent, which could have made an impact on her decision and consent of donating her frozen eggs ... '
		Sensitivity of Reproductive Material Donation	• ' ... It is possible that in the future, there will be consequences which Jane may have to deal with (which Jane couldn't foresee and wasn't explained in the consent forms)'
		Type of Consent	• 'She only verbally told the clinic of her wishes, there doesn't appear to have been any formal agreement signed'
	**Disagree***** **(19)**	Information Disclosure	• 'I don't feel that one can call this informed consent ... she is totally in the dark about any associated risks'<br>• 'Because she has not been given the basic information as to what exactly the eggs will be used for'
		Sensitivity of Reproductive Material Donation	• ' ... Jane's eggs could've been used to study something that goes against her moral beliefs'<br>• 'It could be that they will be used to research something that she fundamentally is not in favour of'
		Type of Consent	• 'She only verbally told the clinic of her wished, there doesn't appear to have been any formal agreement signed'

* *Agree represents mean Likert agreement values of:* >4.5.
** *Neither Agree nor Disagree represents mean Likert agreement values of:* ≤4.5 *and* ≥3.5.
*** *Disagree represents mean Likert agreement values of:* <3.5.

© The Author(s) 2026. Published by Oxford University Press on behalf of European Society of Human Reproduction and Embryology.
This is an Open Access article distributed under the terms of the Creative Commons Attribution License (https://creativecommons.org/licenses/by/4.0/), which permits unrestricted reuse, distribution, and reproduction in any medium, provided the original work is properly cited.
Human Reproduction, 2026, 00, 1–10
https://doi.org/10.1093/humrep/deag007
Original Article
